# Low‐dose radiation prevents type 1 diabetes‐induced cardiomyopathy *via* activation of AKT mediated anti‐apoptotic and anti‐oxidant effects

**DOI:** 10.1111/jcmm.12823

**Published:** 2016-03-15

**Authors:** Fangfang Zhang, Xiufei Lin, Lechu Yu, Weihua Li, Dingliang Qian, Peng Cheng, Luqing He, Hong Yang, Chi Zhang

**Affiliations:** ^1^ Chinese‐American Research Institute for Diabetic ComplicationsWenzhou Medical UniversityWenzhouChina; ^2^ Ruian Center of Chinese‐American Research Institute for Diabetic ComplicationsWenzhou Medical UniversityWenzhouChina; ^3^ Department of Pathologythe Third Affiliated Hospital of Wenzhou Medical UniversityWenzhouChina; ^4^ Department of Inspectionthe Third Affiliated Hospital of Wenzhou Medical UniversityWenzhouChina

**Keywords:** diabetic cardiomyopathy, low‐dose radiation, cardiac hypertrophy remodelling, oxidative stress, apoptosis

## Abstract

We investigated whether low‐dose radiation (LDR) can prevent late‐stage diabetic cardiomyopathy and whether this protection is because of the induction of anti‐apoptotic and anti‐oxidant pathways. Streptozotocin‐induced diabetic C57BL/6J mice were treated with/without whole‐body LDR (12.5, 25, or 50 mGy) every 2 days. Twelve weeks after onset of diabetes, cardiomyopathy was diagnosed characterized by significant cardiac dysfunction, hypertrophy and histopathological abnormalities associated with increased oxidative stress and apoptosis, which was prevented by LDR (25 or 50 mGy only). Low‐dose radiation‐induced cardiac protection also associated with P53 inactivation, enhanced Nrf2 function and improved Akt activation. Next, for the mechanistic study, mouse primary cardiomyocytes were treated with high glucose (33 mmol/l) for 24 hrs and during the last 15 hrs bovine serum albumin‐conjugated palmitate (62.5 μmol/l) was added into the medium to mimic diabetes, and cells were treated with LDR (25 mGy) every 6 hrs during the whole process of HG/Pal treatment. Data show that blocking Akt/MDM2/P53 or Akt/Nrf2 pathways with small interfering RNA of akt, mdm2 and nrf2 not only prevented LDR‐induced anti‐apoptotic and anti‐oxidant effects but also prevented LDR‐induced suppression on cardiomyocyte hypertrophy and fibrosis against HG/Pal. Low‐dose radiation prevented diabetic cardiomyopathy by improving cardiac function and hypertrophic remodelling attributed to Akt/MDM2/P53‐mediated anti‐apoptotic and Akt/Nrf2‐mediated anti‐oxidant pathways simultaneously.

## Introduction

Diabetic cardiomyopathy is one of the most severe complications of diabetes and is characterized by cardiac remodelling including cardiac hypertrophy (CH) and pro‐fibrotic changes associated with cardiac dysfunction 1, 2, 3. The pathogenesis of diabetic cardiomyopathy is complex and is chiefly thought to arise from diabetes‐induced apoptosis and oxidative stress. Dead cardiac cells are replaced by an extracellular matrix which impairs myocardial contractility, increases interstitial fibrosis, and leads to cardiac remodelling and dysfunction 4, 5, 6. Also, oxidative stress as a result of the induction of mitochondrial‐derived reactive oxygen species (ROS), activates diverse hypertrophic signalling kinases and transcription factors to trigger cardiomyocyte dysfunction, DNA mutation and irreversible cell damage that enhances apoptosis 7. Therefore, to prevent diabetic cardiomyopathy, an ideal therapy may simultaneously suppress oxidative stress and apoptosis.

Most of the currently used drugs against diabetic cardiomyopathy in the clinics need to be metabolized and excreted from the liver and kidney, which will increase their load. Growing evidence demonstrate that low dose radiation (LDR), less than 100 mGy for low linear energy transfer, as an invasive approach induces hormesis effect including suppressing gene mutations, enhancing immunity and prolonging the life span 8, 9. Furthermore, previous studies indicate that LDR prevents diabetic nephropathy by suppressing dyslipidaemia, inflammation and oxidative stress 10, 11, 12, 13. Reports also confirm that LDR has anti‐apoptotic effects in testis and hippocampal neuronal cells in diabetic rodents. How does the effect of LDR on diabetic cardiomyopathy is still unclear. Our preliminary work indicates that LDR can prevent cardiac damage *via* suppressing inflammation during early stages of diabetes 14. But other studies also demonstrated that significant inflammation was normally observed in the short‐term rather than long‐term diabetes 15, 16, 17, 18. Therefore, if LDR induces cardiac protection in long‐term diabetic mice, other protective mechanisms must exist instead of anti‐inflammation.

The protein kinase B/Akt is a family of serine/threonine protein kinase. Strong evidence demonstrated that activation (phosphorylation) of Akt positively mediated both cellular anti‐apoptotic and anti‐oxidative functions in the heart simultaneously through MDM2/P53 and GSK3β‐Fyn‐Nrf2 pathways respectively 19, 20, 21, 22, 23. Under diabetic condition Akt activation and Nrf2 expression was decrease which associated with cardiac damage 24, 25, 26. Our previous study indicated that exposure to LDR significantly prevented diabetes‐induced inhibition of renal Akt actication and Nrf2 expression. Whether Akt‐mediated MDM2/P53 and GSK3β‐Fyn‐Nrf2 pathways were also involved in LDR‐induced cardiac protection is still unclear.

To study this mechanism, we established type 1 diabetic mice models *via* multiple treatments with low‐dose streptozotocin (STZ, ip) 27. We then treated animals with whole‐body LDR and measured cardiac effects, specifically, CH, fibrosis and cardiac dysfunction, apoptosis and oxidative stress.

## Materials and methods

### Ethics statement

The animal experiments were performed conform the NIH guidelines (Guide for the care and use of laboratory animals). The protocol was approved by the Committee on the Ethics of Animal Experiments of the Wenzhou Medical University, Zhejiang, China. All surgery was performed under anaesthesia induced by intraperitoneal injection of 1.2% 2,2,2‐Tribromoethanol (Avertin; Sigma‐Aldrich, St. Louis, MO, USA) at the dose of 0.2 ml/10 g bw and all efforts were made to minimize suffering of the experimental animals.

### Establishment of type 1 diabetic mouse model

Eight weeks old, male C57BL/6J mice, were purchased from the Experimental Animal Center of Beijing University (Beijing, China). Mice received i.p. injection of multiple low‐dose STZ (Sigma‐Aldrich) at 50 mg/kg/day for five consecutive days to induced the type 1 diabetes (see Data S1).

### Whole‐body low dose rate X‐ray radiation on mice

Diabetic and non‐diabetic mice were received whole‐body LDR at 12.5, 25 or 50 mGy every 2 days for 12 weeks respectively (see Data S1).

### Cardiomyocytes isolation, culture and LDR treatments and siRNA

Adult mouse cardiomyocytes were isolated as described previously 28. cardiomyocytes were treated with specific siRNAs against *akt1, nrf2* and *p53* with or without LDR (25 mGy), followed by high glucose (33 mmol/l) treatment for 24 hrs and the addition of palmitate (62.5 μmol/l) during the last 15 hrs (see Data S1).

### Echocardiography

Cardiac function and BP were measured by echocardiography and tail‐cuff manometry respectively 27, 29 (see Data S1).

### Morphological examination of cardiac myocardium

Paraffin sections of myocardium from the mice in each group were stained with haematoxylin and eosin and Sirius‐red for the detection of morphological changes or collagen accumulation (fibrosis), respectively, as described previously 14, 27 (see Data S1).

### Terminal deoxynucleotidyl transferase‐mediated dUTP nick end labelling staining

For terminal deoxynucleotidyl transferase‐mediated dUTP nick end labelling (TUNEL) staining, slides were stained with the ApopTag Peroxidase *in situ* Apoptosis Detection Kit (Chemicon, Temecula, CA, USA) 30 (see Data S1).

### Detection of caspase‐3 activity

Caspase‐3 activation was evaluated by detecting caspase‐3 activity as described before 31 (see Data S1).

### Assaying lipid oxidation

A thiobarbituric acid assay was used to measure relative malondialdehyde (MDA) production as an index of lipid peroxidation, as described previously 32 (see Data S1).

### Measurement of ROS generation

Reactive oxygen species generation of cardiomyocyte was examined using the intracellular ROS assay kit (see Data S1).

### Nuclei isolation

Nuclei of the cardiomyocytes from both *in vivo* and *in vitro* studies were isolated using nuclei isolation kit (NUC‐ 201; Sigma‐Aldrich) as previously 23 (see Data S1).

### Western blotting assay

Western blot was performed as described in our previous studies 10, 11 (see Data S1).

### RNA isolation and real‐time quantitative polymerase chain reaction

RNA isolation and real‐time quantitative PCR was performed as described in our previous studies 10, 11 (see Data S1).

### Statistical analysis

Data were collected from eight mice per group, or three replicates of cell‐culture experiments, which presented as mean ± S.D. One‐way anova was used to determine general differences, followed by a post‐hoc Tukey's test for the difference between groups, using Origin 7.5 software for laboratory data analysis and graphing. Statistical significance was considered *P* < 0.05.

## Results

### Effect of LDR on hyperglycaemia in type 1 diabetic mice

The blood glucose levels were similar among groups before induction of diabetes by STZ. Five days after the last dose of STZ, the blood glucose levels were measured. Once hyperglycaemia was diagnosed, diabetic mice and age‐matched control mice were exposed or shamed to LDR either at 12.5 mGy, single 25 mGy or 50 mGy. Figure S1 showed that 5 days after the last STZ injection, the blood glucose level was significantly elevated in the mice of diabetic group (>12 mmol/l, around 17 mmol/l), indicating that the type 1 diabetic mice models were successfully established. Addtionally, the blood glucose levels of diabetic mice were further elevated 12 weeks later (around 25 mmol/l). However, Blood glucose levels in DM/25 mGy and DM/50 mGy group were kept at plateau levels (around 16 mmol/l) after 12 weeks exposure to LDR and showed a statistical difference from those in the DM groups (Fig. S1).

### Exposure to LDR prevented type 1 diabetes‐induced cardiac dysfunction

Using Echo examination (Table 1), we measured diabetic mouse cardiac function and noted progressive increase in LVID;s, IVS;d and LVPW;d and progressive decrease in LVPW;s, EF% and FS%. Exposure to LDR at 25 or 50 mGy was cardioprotective but neither radiation dose was statistically significantly different between the two groups. However, similar protective effect was not found in diabetic mice exposed to 12.5 mGy.

**Table 1:** Effect of LDRs on cardiac function in type 1 diabetic mice

	Con	25 mGy	DM	DM/12.5 mGy	DM/25 mGy	DM/50 mGy
LVID;d (mm)	3.61 ± 0.05	3.63 ± 0.1	3.98 ± 0.04^a^	3.93 ± 0.1	3.75 ± 0.04^a^^,^ b	3.73 ± 0.06^a^^,^ b
LVID;s (mm)	1.69 ± 0.16	1.56 ± 0.13	2.27 ± 0.15^a^	2.19 ± 0.13	1.81 ± 0.11^a^^,^ b	1.75 ± 0.13^b^
IVS;d (mm)	0.73 ± 0.03	0.76 ± 0.01	0.81 ± 0.01^a^	0.79 ± 0.02	0.74 ± 0.02^a^^,^ b	0.72 ± 0.01^b^
IVS;s (mm)	1.14 ± 0.02	1.11 ± 0.04	0.89 ± 0.05	0.94 ± 0.02	1.01 ± 0.04	1.11 ± 0.02
LVPW;d (mm)	0.82 ± 0.01	0.84 ± 0.03	1.21 ± 0.03^a^	1.14 ± 0.05	1.01 ± 0.01^a^^,^ b	0.94 ± 0.03^a^^,^ b
LVPW;s (mm)	1.82 ± 0.13	1.82 ± 0.09	1.31 ± 0.1^a^	1.4 ± 0.2	1.59 ± 0.14^a^^,^ b	1.74 ± 0.11^a^^,^ b
%EF (%)	89.11 ± 1.32	88.35 ± 1.09	63.89 ± 2.02^a^	68.43 ± 3.59	76.35 ± 3.31^a^^,^ b	80.89 ± 2.02^a^^,^ b
%FS (%)	61.22 ± 1.16	60.68 ± 1.21	39.48 ± 1.07^a^	44.22 ± 5.16	52.68 ± 4.21^a^^,^ b	55.93 ± 1.07^a^^,^ b
LV mass (mg)	92.23 ± 4.12	89.68 ± 3.69	110.58 ± 6.46^a^	107.43 ± 4.97	103.44 ± 2.85^a^^,^ b	98.49 ± 6.32^a^^,^ b
LV mass‐C (mg)	73.43 ± 2.11	75.37 ± 2.74	88.47 ± 3.56^a^	82.75 ± 1.13	78.69 ± 1.02^a^^,^ b	76.45 ± 2.22^a^^,^ b

a *P* < 0.05 *versus* the control group.

b *P* < 0.05 *versus* the DM group.

Data were presented as means ± S.E.M. *n* = 8 in each group.

### LDR protected the heart from diabetes‐induced hypertrophic remodelling, morphological abnormalities and fibrosis

**Figure 1:**
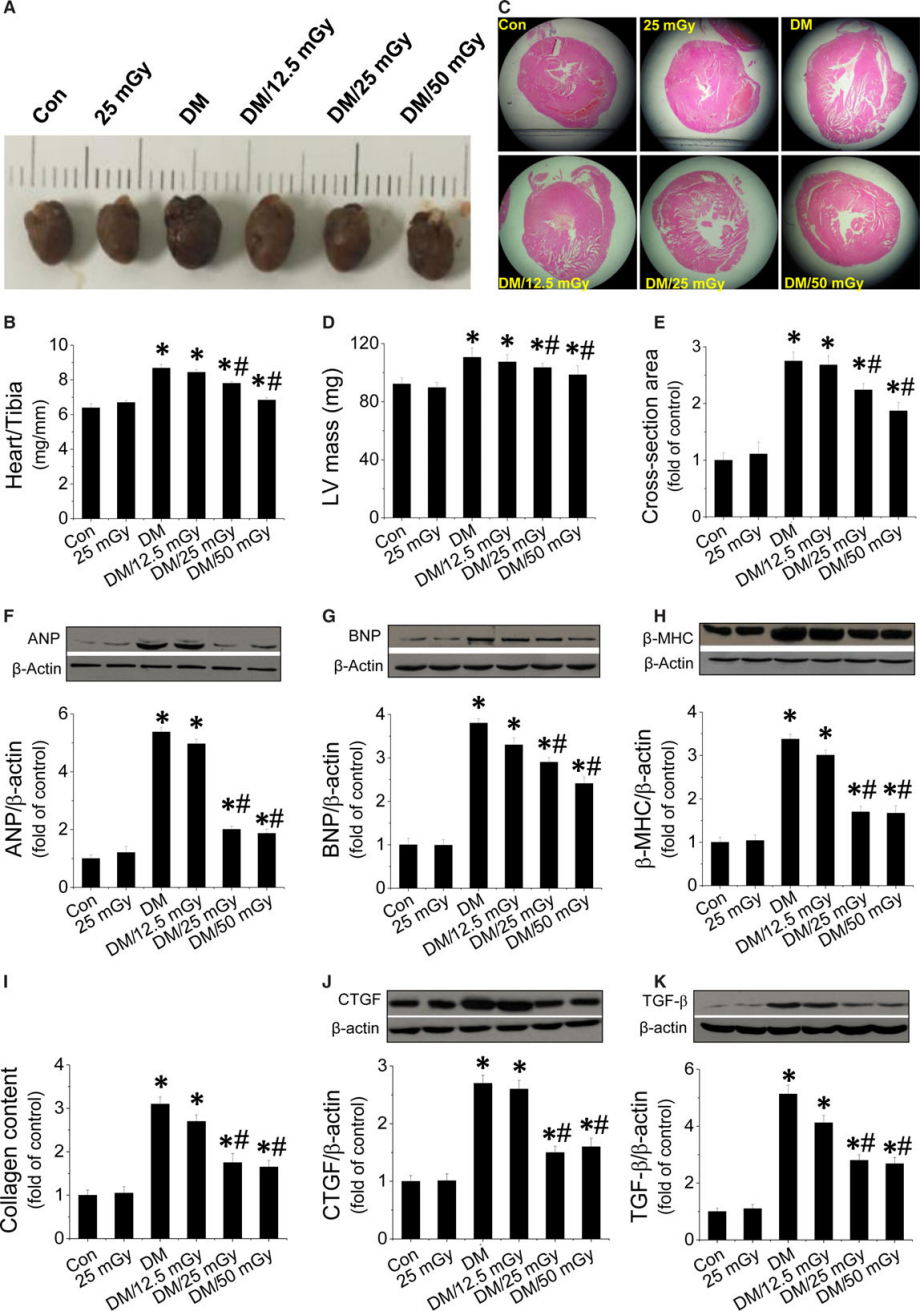
Effect of LDR on diabetes‐induced CH, and fibrosis. Diabetic and age‐matched mice were exposed to LDR at 12.5, 25 or 50 mGy every 2 days for 12 weeks. CH was evaluated by examining the heart size (**A**), the ratio of heart weight to tibia length (HW/BW (**B**), the cross‐section of cardiac chambers (**C**), the LV mass (**D**) and cardiomyocyte size (**E**), as well as the expression of hypertrophic markers including cardiac ANP (**F**), BNP (**G**) and β‐MHC (**H**). Fibrosis was evaluated by measuring collagen content (**I**), expression of fibrotic markers such as CTGF (**J**) and TGF‐β (**K**) in diabetic hearts with Western blot. Data are presented as means ± S.D., *n* = 8/group. *P < 0.05 *versus* the control (Con) group; ^#^ *P* < 0.05 *versus* diabetic (DM) group.

**Figure 2:**
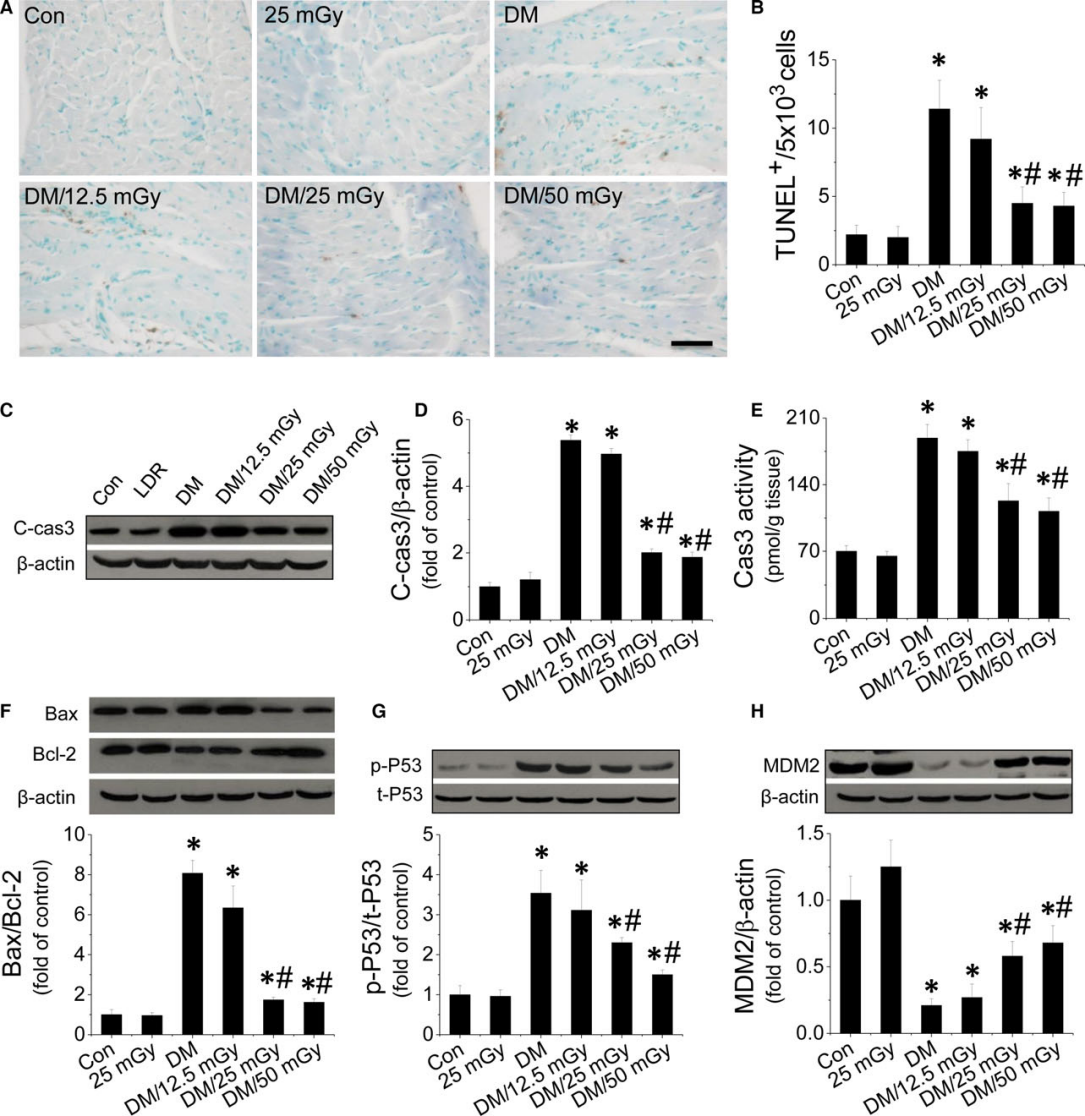
Effect of LDR on mitochondrial pathway‐mediated apoptosis in diabetic hearts. Apoptosis was measured by TUNEL staining (**A**), followed by quantitative analysis of TUNEL‐positive cells (**B**). Cardiac apoptosis was confirmed by measuring cleaved‐caspase‐3 expression (**C** and **D**) and caspase‐3 activity (**E**) with Western blot and ELISA. Expression of Bax and Bcl‐2, markers of the mitochondrial death pathway, were measured and the ratio of Bax/Bcl‐2 is given (**F**). P53 activity (**G**), an inducer of the mitochondrial death pathway, and expression of its negative regulator, MDM2 (**H**) were measured. Data are presented as means ± S.D., *n* = 8/group. *P < 0.05 *versus* the Con group; ^#^ *P* < 0.05 *versus* the DM group.

A significant increase in the size of heart (Fig. 1A), as well as the ratio of heart weight to tibia length was observed in non‐treated diabetic mice, suggesting possible induction of CH (Fig. 1B). The CH was further confirmed by the size increase in cardiac chamber of diabetic mice (Fig. 1C). Additionally, we also observed increased LV mass (Fig. 1D) and cardiomyocyte size (Fig. 1E, Fig. S2A) in diabetic hearts. Consistently, expression of hypertrophic markers including ANP (Fig. 1F), BNP (Fig. 1G) and β‐MHC (Fig. 1H) were strongly increased in diabetic hearts. However, exposure to LDR at 25 or 50 mGy (not 12.5 mGy), prevented these increased hypertrophic parameters (Fig. 1A–H and Fig. S2A). Moreover, in diabetic hearts, morphological abnormalities including focal cell necrosis, disorganized array of myocardial structure and myofibrillar discontinuation were observed under haematoxylin and eosin staining (Fig. S2A). Meanwhile, Sirius‐red staining for fibrosis confirmed that diabetes caused significant collagen accumulation in both the perivascular and the interstitial tissues (Fig. S2B, Fig 2I). Consistent with Sirius‐red staining, expressions of fibrotic markers at the molecular level including connective tissue growth factor (CTGF) (Fig. 2J) and transforming growth factor (TGF)‐β (Fig. 2K) were significantly up‐regulated in diabetic hearts. However, all the fibrotic effects induced by diabetes were remarkably prevented by exposure to LDR at 25 or 50 mGy, but not at 12.5 mGy.

### LDR induced anti‐apoptotic effect in diabetic hearts

TUNEL assay was performed on cardiac tissues to measure apoptosis. Increased apoptosis (*i.e*. TUNEL‐positive cells; Fig. 2A and B) in diabetic hearts was observed compared to non‐diabetic hearts. However, the increased apoptosis was significantly suppressed by exposure to LDR at 25 or 50 mGy. To confirm the inhibitory effect of LDR on diabetes‐induced cardiac apoptosis, caspase‐3 activation and active or cleaved product were measured by enzymatic assay and Western blot. Data show that both cleaved caspase‐3 content and caspase‐3 activity strongly increased in the diabetic hearts (Fig. 2C–E). In contrast, exposure to LDR at 25 or 50 mGy, but not 12.5 mGy, significantly prevented cardiac apoptosis (Fig. 2A–E). Meanwhile analysis of the Bax/Bcl‐2 ratio as a mitochondrial cell death pathway revealed a synergistic increase in Bax/Bcl‐2 ratios in diabetic hearts (Fig. 2F). Additionally, we also investigated the activity of cardiac P53 (Fig. 2G), which is an upstream inducer of the mitochondrial death pathway. The activity (phosphorylation) of cardiac P53 was significantly enhanced in the diabetic hearts and this was accompanied by a significant decrease in cardiac expression of MDM2 (Fig. 2G and H), a negative regulator of P53. After exposure to LDR at 25 or 50 mGy, cardiac P53 activation was strongly inhibited and this was a consistent finding with regard to increased expression of cardiac MDM2 (Fig. 2G and H).

### LDR prevented diabetes‐induced oxidative stress in hearts associated with enhanced Nrf2 expression and function

**Figure 3:**
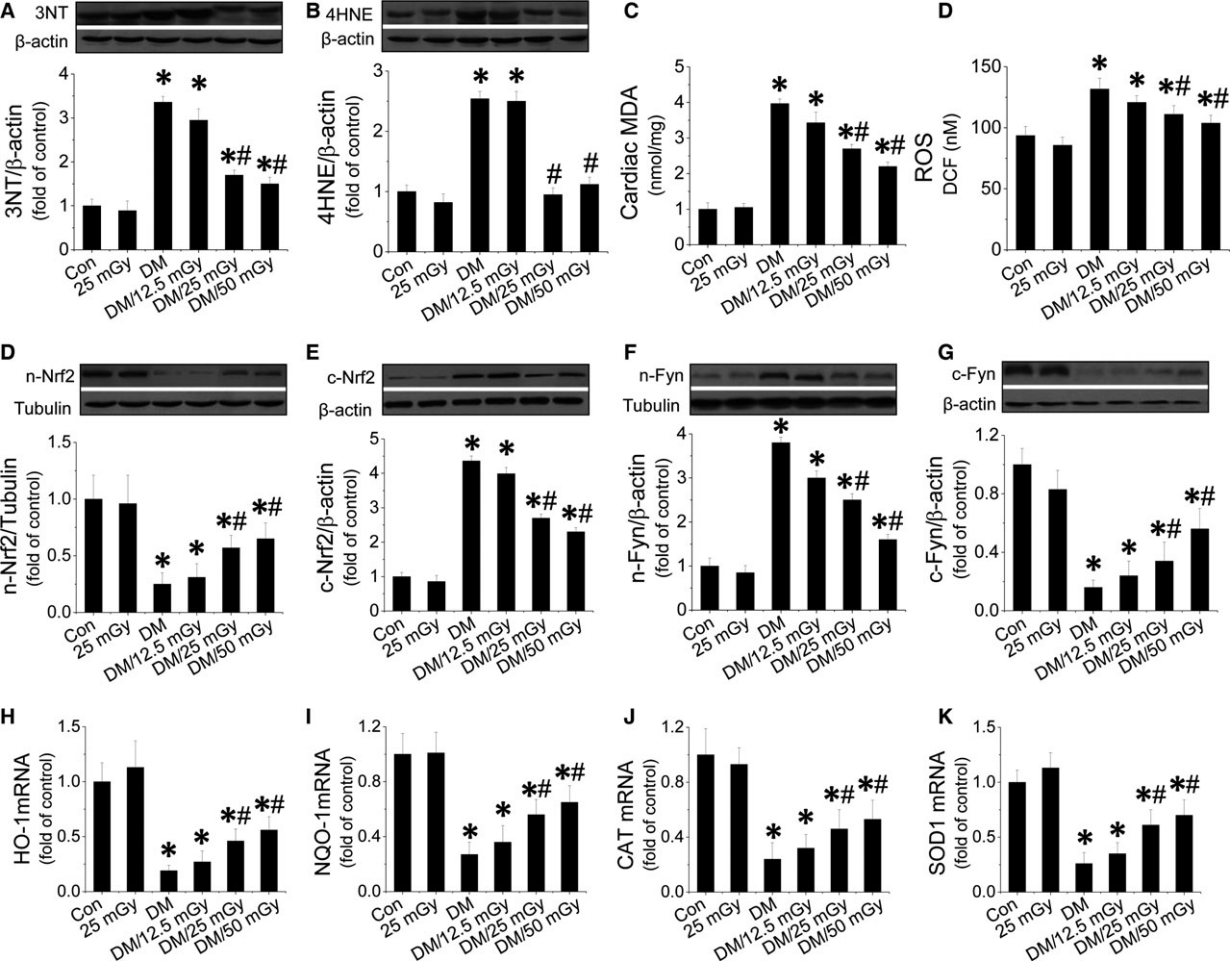
Effect of LDR on diabetes‐induced oxidative stress in the diabetic hearts. Cardiac tissues from all four groups were collected to measure cardiac oxidative stress. Expression of the nitrosative damage marker 3‐NT (**A**) and the oxidative marker (**B**) were measured with Western blot. MDA (**C**) and cardiac ROS was measured (**D**) with ELISA. The translocation between the nuclei and cytosol of Nrf2 (**D** and **E**) or Fyn (**F** and **G**) was evaluated by measuring protein of each in the nuclei and cytosol respectively. Nrf2 function was measured by quantifying expression of Nrf2 downstream genes at the mRNA level including HO‐1 (**H**), NQO1 (**I**), CAT (**J**), SOD‐1 (**K**) with real‐time PCR. Data are presented as means ± S.D., *n* = 8/group. *P < 0.05 *versus* the Con group; ^#^ *P* < 0.05 *versus* the DM group.

We investigated the effect of LDR on oxidative stress in the diabetic hearts as measured by 3‐NT as an index of nitrosative damage (Fig. 3A), and 4‐HNE (Fig. 3B) and MDA (Fig. 3C) as a classic oxidative damage markers. These were significantly increased in the diabetic hearts. Exposure to LDR at 25 or 50 mGy significantly decreased the contents of the above oxidative markers in the diabetic hearts. Because oxidative stress is because of the imbalance between ROS production and scavenging, we measured cardiac ROS in each treatment group and found that LDR at 25 or 50 mGy strongly inhibited ROS production in the diabetic hearts (Fig. 3D). Nrf2 is an important cellular defence mechanism against oxidative stress, which can translocate to the nucleus from the cytosol and induce transcription of genes encoding various anti‐oxidants. Diabetes inhibited Nrf2 nuclear translocation (Fig. 3D and E), and this was associated with opposing translocation of Fyn, a negative regulator of Nrf2 (Fig. 3F and G). However, the impaired nuclear translocation of Nrf2 was remarkably reversed after exposure to LDR at 25 or 50 mGy. Next, we further studied the transcriptional function of Nrf2 by measuring its downstream anti‐oxidant expression including HO‐1 (Fig. 3H, Fig. S3A), NQO1 (Fig. 3I, Fig. S3B), CAT (Fig. 3J, Fig. S3C), SOD‐1 (Fig. 3K, Fig. S3D) at the mRNA and protein levels. These anti‐oxidants were strongly suppressed in the diabetic hearts. However, exposure to LDR at 25 or 50 mGy reversed mRNA and protein expression of these markers.

### LDR prevented inactivation of the PI3K‐AKT‐GSK3β signalling pathway in the diabetic hearts

Diabetes‐induced cardiac apoptosis and oxidative stress are considered to be associated with inactivation of the PI3K‐AKT‐GSK‐3β pathway 24, 33, 34, which was confirmed here, as demonstrated by a significant decrease in the expression of PI3K (Fig. S4A) and the phosphorylation of AKT (Fig. S4B) and its downstream target GSK‐3β (Fig. S4C) in the diabetic hearts. These expression patterns were significantly reversed by LDR at 25 or 50 mGy.

### LDR prevented HG/Pal‐induced hypertrophic and fibrotic effects in primary cardiomyocytes associated with suppression of oxidative stress and apoptosis

**Figure 4:**
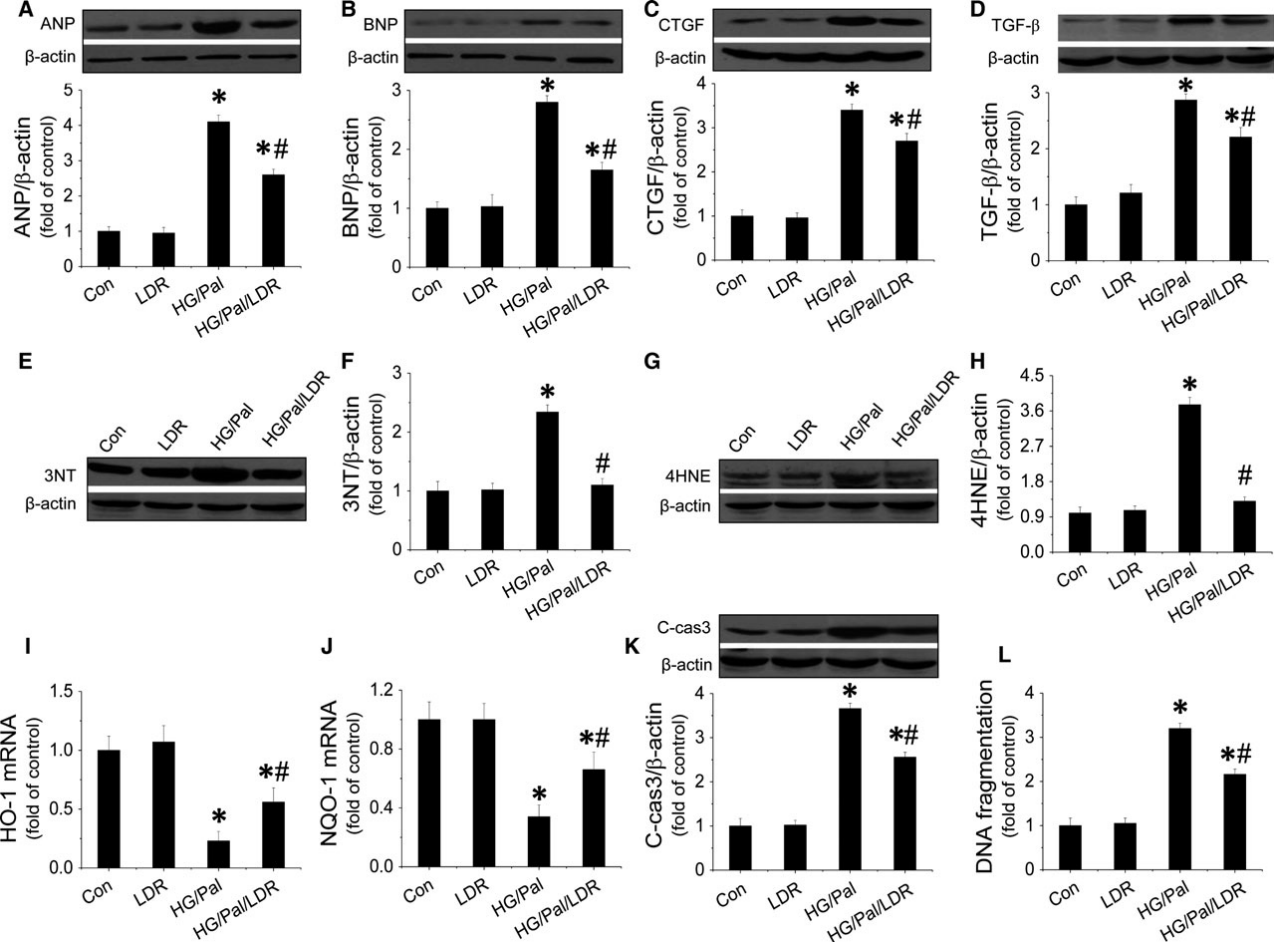
LDR prevented hypertrophy, fibrosis, oxidative stress and apoptosis in HG/Pal‐treated primary cardiomyocytes. Primary cardiomyocytes from adult mice were isolated and treated with high glucose (33 mmol/l) for 24 hrs associated with palmitate (62.5 μmol/l) during the last 15 hrs. Cells were exposed to LDR at 25 mGy every 6 hrs initiated just before high‐glucose treatment. Western blot assay was applied to identify cardiomyocyte hypertrophy by measuring expressions of ANP (**A**) and BNP (**B**); fibrosis in cardiomyocytes by measuring expressions of CTGF (**C**) and TGF‐β (**D**); oxidative stress in cardiomyocytes by examining the expression 3‐NT (**E** and **F**), 4‐HNE (**G** and **H**), HO‐1 mRNA (**I**) and NOQ‐1 mRNA (**J**); and cell apoptosis by quantifying expression of cleaved‐caspase‐3 (**K**) and DNA fragmentation (**L**). Data are presented as means ± S.D., *n* = 8/group. *P < 0.05 *versus* the Con group; ^#^ *P* < 0.05 *versus* the DM group.

*In vivo* data suggest that LDR was protective against diabetic cardiomyopathy but how this occurs is unclear. Thus, primary cultured mouse cardiomyocytes were treated with HG (33 mmol/l) and Pal (62.5 μmol/l). Markers of cardiomyocyte hypertrophy, fibrosis, oxidative stress and apoptosis were measured with Western blot or Real‐time PCR. Data show that HG/Pal induced cardiomyocyte hypertrophy by up‐regulating ANP (Fig. 4A) and BNP (Fig. 4B) expression; induced fibrosis by increasing CTGF (Fig. 4C) and TGF‐β expression (Fig. 4D); induced oxidative stress by increasing expression of 3‐NT (Fig. 4E and F) and 4‐HNE (Fig. 4G and H) as well as decreasing HO‐1 (Fig. 4I) and NQO‐1 mRNA (Fig. 4J). Cardiomyocyte apoptosis was induced by increased cleaved‐caspase‐3 expression (Fig. 4K) and DNA fragmentation (Fig. 4L). In contrast, these abnormalities were remarkably inhibited by LDR at 25 mGy. Additionally, we also confirmed that LDR‐induced anti‐oxidant effects were associated with improving Nrf2 nuclear translocation (Fig. S5A and B), as well opposing translocation of Fyn (Fig. S5C and D). LDR‐induced anti‐apoptotic effects in HG/Pal‐treated primary cardiomyocytes were associated with inactivation of P53 (Fig. S5E), as well as increased MDM2 expression (Fig. S5F). Furthermore, we also found that HG/Pal treatment significantly inhibited the phosphorylation of Akt (Fig. S5G) and GSK‐3β (Fig. S5H) in cardiomyocytes, and this was reversed by exposure to LDR at 25 mGy.

### Akt‐mediated LDR‐induced protection in HG/Pal‐treated cardiomyocytes

**Figure 5:**
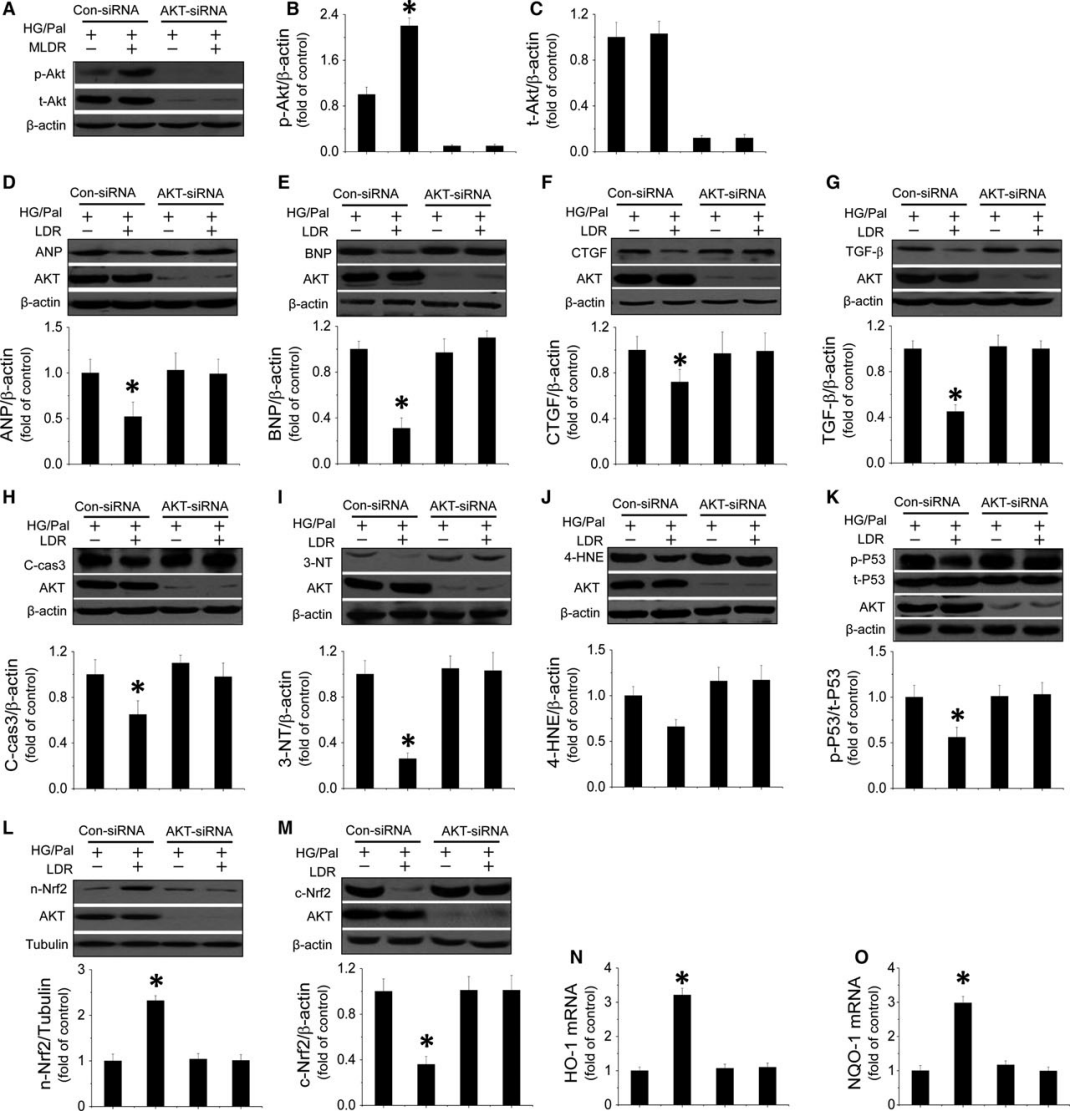
Akt mediates LDR‐induced antihypertrophic and antifibrotic effects against HG/Pal associated with suppression of P53‐induced apoptosis and improvement of Nrf2 translocation and transcriptional function. Primary cardiomyocytes were transfected with either negative control sense siRNA or mouse Akt antisense siRNA. Western blot was used to measure Akt phosphorylation (**A** and **B**) and expression (**A** and **C**). Expression of ANP (**D**) and BNP (**E**) as markers of hypertrophy; CTGF (**F**) and TGF‐β (**G**) as markers of fibrosis were measured by Western blot assay. Expression of cleaved‐caspase3 (**H**) and 3‐NT (**I**), 4‐HNE (**J**) reflecting apoptosis or oxidative stress were quantified. To measure mediators of both apoptotic and oxidative pathways induced by HG/Pal treatment, P53 activity (**K**) and Nrf2 translocation (**L** and **M**) and transcriptional function (**N** and **O**) were evaluated by Western blot. Data are presented as means ± S.D., *n* = 8/group. *P < 0.05 *versus* the Con group; ^#^ *P* < 0.05 *versus* the DM group.

Although we found that LDR at 25 mGy‐induced cardiac protection *in vitro* was associated with activation of Akt, whether this protection is mediated by Akt remains unclear. Therefore, the direct role of Akt in the LDR‐induced therapeutic response was tested by knocking down Akt expression with its siRNA. We noted that Akt siRNA effectively reduced Akt phosphorylation (Fig. 5A and B) and expression (Fig. 5A and C) in HG/Pal‐treated cardiomyocytes with/without exposure to LDR at 25 mGy. Next, we confirmed that exposure to LDR at 25 mGy significantly prevented HG/Pal‐induced hypertrophy and fibrosis characterized by down‐regulation of ANP (Fig. 5D), BNP (Fig. 5E), CTGF (Fig. 5F) and TGF‐β expression (Fig. 5G) in con‐siRNA‐treated cells, but not in Akt‐siRNA‐treated cells. Similarly, knockdown of Akt suppressed LDR‐induced down‐regulation of the apoptotic marker, cleaved‐caspase‐3 (Fig. 5H) and the oxidative marker, 3‐NT (Fig. 5I) and 4‐HNE expression (Fig. 5J). Furthermore, Akt knockdown also suppressed LDR‐induced inactivation of P53 (Fig. 5K), and Nrf2 nuclear translocation (Fig. 5L and M) and its transcriptional function (Fig. 5N and O). Therefore, we confirmed that LDR at 25 mGy prevented diabetes‐induced hypertrophy and fibrosis in HG/Pal‐treated cardiomyocytes attributed to Akt‐mediated anti‐apoptotic and anti‐oxidant pathways.

### LDR protected cardiomyocytes against HG/Pal treatment partially by suppression of the MDM2‐P53‐mediated apoptotic signalling pathway

**Figure 6:**
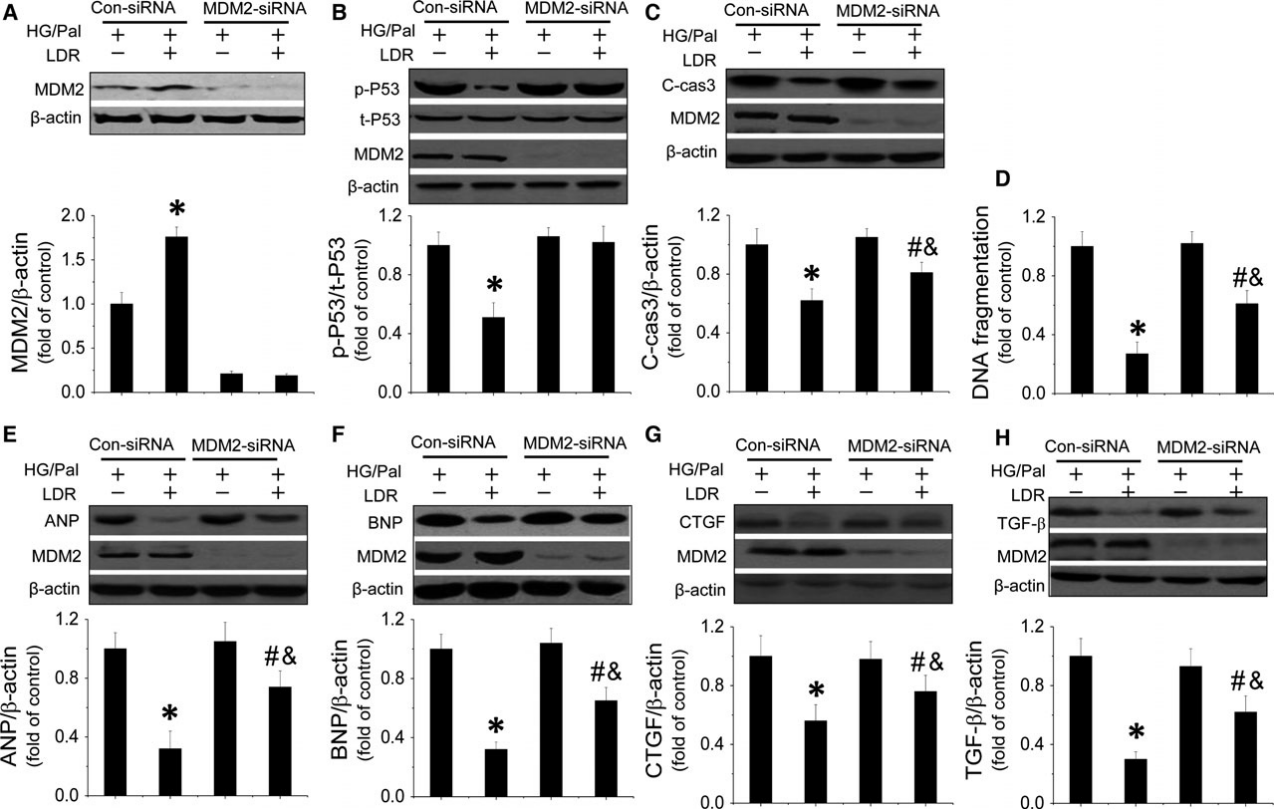
MDM2/P53‐mediated anti‐apoptotic pathway is involved in LDR‐induced cardiac protection *in vitro*. TO evaluate the relationship between MDM2/P53‐mediated anti‐apoptotic effects and LDR‐induced cardiac protection *in vitro* against HG/Pal. Primary cardiomyocytes were transfected with either negative control sense siRNA or mouse MDM2 antisense siRNA using Lipofectamine TM 2000 transfection reagent for 48 hrs. Transfection was followed by treatment with HG/Pal with/without exposure to LDR at 25 mGy. Western blot was used quantify P53‐mediated apoptosis by measuring MDM2 expression (**A**) P53 phosphorylation and cleaved‐caspase3 expression (**B** and **C**). Meanwhile another apoptotic marker, DNA fragmentation, was measured by ELISA (**D**). Additionally, expression of hypetrophic markers, ANP (**E**) and BNP (**F**) and fibrotic markers, CTGF (**G**) and TGF‐β (**H**) were measured by Western blot. Data are presented as means ± S.D., *n* = 8/group. *P < 0.05 *versus* the Con group; ^#^ *P* < 0.05 *versus* the DM group.

Whether Akt‐mediated inactivation of the MDM2‐P53 signalling pathway participated in this protection was tested by knocking down MDM2 expression with its siRNA. We first demonstrated that MDM2 siRNA effectively reduced MDM2 expression in cardiomyocytes (Fig. 6A), which also completely abolished LDR‐induced inhibition of P53 phosphorylation (Fig. 6B) and the following cleaved‐caspase‐3 expression and DNA fragmentation (Fig. 6C and D). Further study demonstrated that MDM2 knockdown reduced the ability of LDR to reduce ANP, BNP, CTGF and TGF‐β expression in HG/Pal‐treated cardiomyocytes (Fig. 6E–H).

### LDR‐induced cardiac protection against HG/Pal treatment *in vitro* partially by improving the Nrf2‐mediated anti‐oxidant signalling pathway

**Figure 7:**
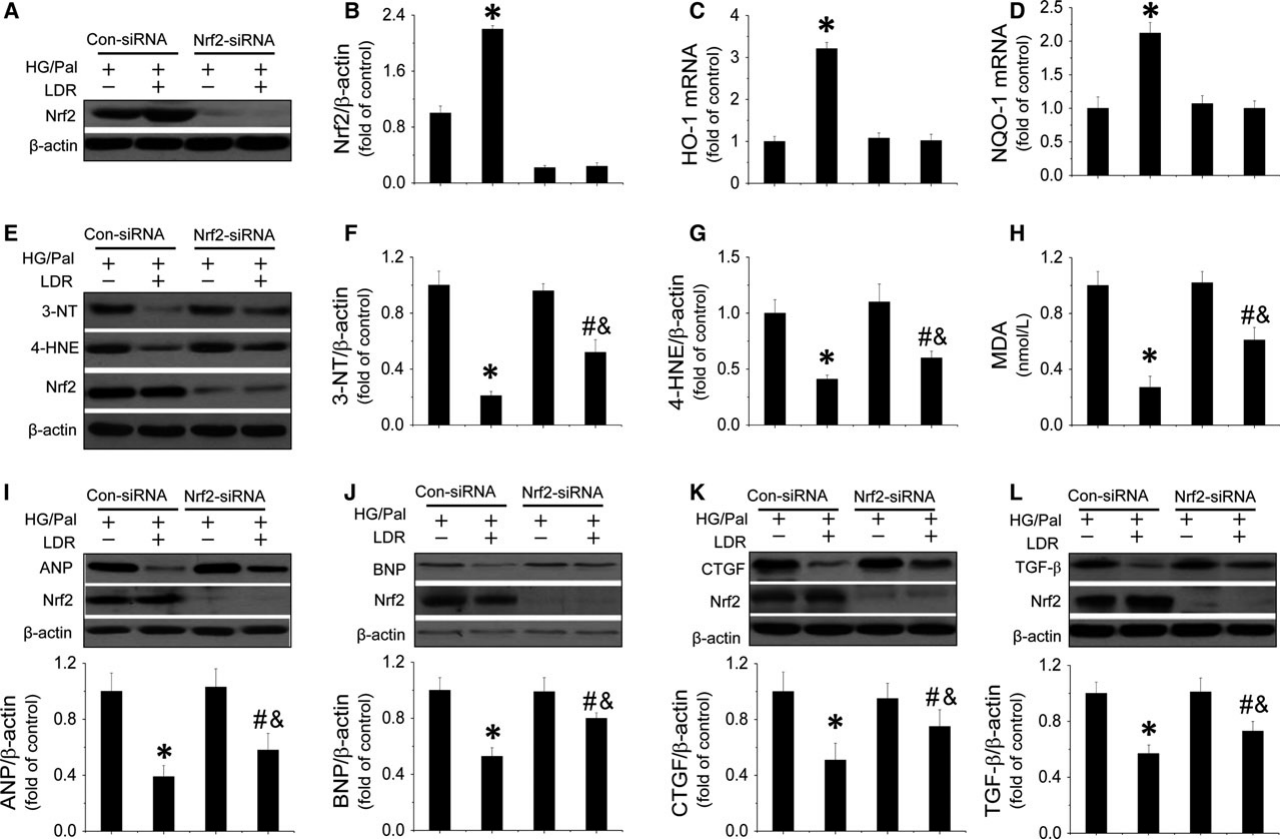
Nrf2‐mediated anti‐apoptotic pathway is involved in LDR‐induced cardiac protection *in vitro*. To understand the relationship between Nrf2‐mediated anti‐oxidant effect and LDR‐induced cardiac protection in against HG/Pal *in vitro*. Primary cardiomyocytes were transfected with either negative control sense siRNA or mouse MDM2 antisense siRNA using Lipofectamine TM 2000 transfection reagent for 48 hrs. Transfection was followed by treatment with HG/Pal with and without exposure to LDR at 25 mGy. Firstly, the effect of nrf2 silencing was evaluated by comparing Nrf2 expression (**A** and **B**) and its transcriptional function (**C** and **D**). Meanwhile expressions of oxidative markers including 3‐NT (**E** and **F**), 4‐HNE (**E** and **G**) and MDA (**H**) were also measured by Western blot or ELISA. Additionally, expression of hypetrophic markers, ANP (**E**) and BNP (**F**) and expression of fibrotic markers, CTGF (**G**) and TGF‐β (**H**) were quantified. Data are presented as means ± S.D., *n* = 8/group. *P < 0.05 *versus* the Con group; ^#^ *P* < 0.05 *versus* the DM group.

Whether Nrf2 is essential for cardiac protection provided by LDR was investigated by knocking down Nrf2 expression with its siRNA, which also can attenuate the nuclear translocation of Nrf2. We found Nrf2 siRNA effectively reduced the expressions of Nrf2 (Fig. 7A and B) and completely inhibited LDR‐induced up‐regulation of HO‐1 and NQO‐1 mRNA in cardiomyocytes (Fig. 7C and D). Meanwhile, knockdown of Nrf2 also remarkably suppressed LDR‐induced inhibition of 3‐NT (Fig. 7E and F), 4‐HNE expression (Fig. 7E and G) and reduced MDA (Fig. 7H). Nrf2 knockdown partially reduced LDR's ability to reduce hypertrophic markers (ANP and BNP expression) (Fig. 7I and J), and fibrotic markers (CTGF and TGF‐β expression) in HG/Pal‐treated cardiomyocytes (Fig. 7K and L).

### The effect of LDR on plasmatic cancer markers level

The oncogenicity is the major concern of LDR's safety during applied in the clinics. Therefore, we measured a range of plasmatic cancer markers in mice among groups. The results was shown in Table 2 that all the cancer markers maintained at low levels in the plasma of healthy mice. Similarly, the above cancer markers in the plasma of either diabetic or LDR‐treated mice also maintained at similarly low levels, which means LDR did not have tumorgenesis ability at the doses of 12.5, 25 or 50 mGy.

**Table 2:** Effect of LDRs on plasma cancer markers in type 1 diabetic mice

	Con	25 mGy	DM	DM/12.5 mGy	DM/25 mGy	DM/50 mGy
AFP (U/l)	8.00 ± 0.67	8.02 ± 0.78	8.10 ± 0.54	8.10 ± 0.83	8.03 ± 0.64	8.06 ± 0.58
CEA (ng/ml)	0.58 ± 0.16	0.57 ± 0.11	0.60 ± 0.15	0.60 ± 0.13	0.58 ± 0.15	0.60 ± 0.13
CA‐125 (U/ml)	3.22 ± 0.56	3.12 ± 0.65	3.30 ± 0.35	3.14 ± 0.68	3.20 ± 0.52	3.16 ± 0.41
CA‐153 (U/ml)	2.54 ± 0.22	2.46 ± 0.24	2.60 ± 0.25	2.56 ± 0.22	2.48 ± 0.24	2.49 ± 0.22
CA‐199 (U/ml)	8.56 ± 2.11	8.32 ± 1.86	8.48 ± 1.95	8.44 ± 1.57	8.33 ± 1.28	8.46 ± 1.60
CA‐724 (U/ml)	1.00 ± 0.13	1.04 ± 0.09	0.97 ± 0.11	1.02 ± 0.12	0.95 ± 0.14	1.06 ± 0.11
NSE (ng/ml)	41.65 ± 5.33	40.12 ± 4.38	43.00 ± 6.28	41.99 ± 5.54	42.64 ± 6.38	41.53 ± 4.68
PSA (ng/ml)	6.65 ± 1.13	6.42 ± 1.21	6.5 ± 1.07	6.32 ± 5.16	6.36 ± 4.21	6.35 ± 1.07
SCCA (μg/l)	0.32 ± 0.08	0.35 ± 0.04	0.34 ± 0.05	0.34 ± 0.03	0.36 ± 0.05	0.33 ± 0.03
Ferritin (ng/ml)	45.56 ± 5.55	46.35 ± 4.73	43.43 ± 5.54	47.26 ± 6.47	46.53 ± 6.30	45.65 ± 3.34

Data were presented as means ± S.E.M. *n* = 8 in each group.

AFP: Alpha foetal protein; CEA: Cancer embryo antigen; CA‐125: Cancer antigen‐125; CA‐153: Cancer antigen‐153; CA‐724: Cancer antigen‐724; NSE: neuron‐specific enolase; PSA: Prostate‐specific antigen; SCCA: Squamous cell carcinoma antigen.

## Discussion

Diabetic cardiomyopathy has been defined as CH, fibrosis and ventricular dysfunction that occur independently of coronary artery disease and hypertension 35. Oxidative stress, apoptosis are regarded as main features of diabetic cardiomyopathy 29, 36, 37. Therefore, an ideal drug to prevent diabetes‐induced cardiomyopathy may need to inhibit oxidative stress and apoptosis simultaneously.

Effects of LDR are distinct from those of moderate‐ or high‐level radiation, which stimulate beneficial effects including cell proliferation, metabolic activity and anti‐inflammatory and anti‐oxidant effects 38, 39, 40, 41, 42. Our previous work suggests that exposure to repetitive LDR (25 mGy/day) induces anti‐inflammatory effects in the hearts at the early stage of diabetes 14. However, whether LDR also induces beneficial effects in cardiomyopathy at the late stage of diabetes remains unclear. If so, whether LDR also induces preventive effects in other key pathogenic events including apoptosis and oxidative stress of diabetic cardiomyopathy apart from inflammation still needs to be investigated.

To confirm that LDR is protective against diabetic cardiomyopathy, a type 1 diabetic mice model was established and mice received LDR exposure at 12.5, 25, 50 mGy, respectively, for 12 weeks. We observed significant cardiac dysfunction and cardiac remodelling including CH and fibrosis in diabetic mice, accompanied by myocardial morphological abnormalities. Accordingly, the mice models of diabetic cardiomyopathy were regarded as successfully established. Interestingly, in the myocardium the symptoms of diabetic cardiomyopathy were significantly prevented by exposure to LDR at medium and high doses (25, 50 mGy) but not at low doses (12.5 mGy) and there were no statistically significant differences between the upper doses of LDR, although visually, there appeared to be more cardiac protective effects offered by 50 mGy.

So, what is the mechanism behind LDR‐protection of the heart from diabetes? As we know, inflammation, apoptosis and oxidative stress are the key pathogeneses of diabetic cardiomyopathy. In the previous study, we have already established that LDR at 25 mGy significantly prevented inflammatory effects in the diabetic heart 14. However, strong evidence demonstrated that the evident inflammation was only observed in short‐term type 1 diabetes rather than in long‐term type 1 diabetes 15, 16, 17, 18, which implied that enhanced enhance inflammation is impossible to be the key pathogengesis of diabetic cardiomyopathy at the late‐stage of type 1 diabetes. Therefore, If LDR can induce preventive effect on diabetic cardiomyopathy, other protective mechanisms rather than anti‐inflammation must exist. Therefore, in the present study, we mainly focused on evaluating the effect of LDR on the other pathogeneses of diabetic cardiomyopathy including apoptosis and oxidative stress. Cardiac apoptosis, examined by TUNEL staining as well as detection of caspase‐3 activation, was significantly induced in the diabetic hearts. However, exposure to LDR prevented diabetes‐induced apoptosis in a dose‐dependent manner as demonstrated by reduction in positive apoptotic cells and caspase‐3 activation. Caspase‐3 is the end‐point apoptotic marker activated by the mitochondrial, endoplasmic reticular or death receptor pathway. Here, increased ratio of Bax to Bcl was strongly, but not completely suppressed, suggesting that LDR prevented diabetes‐induced cardiac cell apoptosis partially because of the inhibition of the mitochondrial pathway. Whether other two apoptotic pathways are also prevented by LDR is unclear. LDR at 25 or 50 mGy, but not 12.5 mGy, significantly prevented oxidative stress in the diabetic hearts, characterized by less ROS production and reduced biomarkers of oxidation, including 3‐NT, 4‐HNE and MDA which are associated with anti‐oxidants including HO‐1, NQO‐1, CAT and SOD‐1. Thus, based on the above evidence, we revealed that anti‐apoptotic and anti‐oxidant stress properties were involved in LDR‐induced cardiac protection against diabetes.

Next, we further explored more mechanisms behind LDR‐induced protective effects in the diabetic hearts. Akt, an effector of PI3K, is a serine/threonine protein kinase that regulates a variety of cellular functions in different tissues 43, 44, 45. The PI3K/Akt signalling pathway is known to mediate beneficial cardiac effects including improvement of cardiac growth, myocardial angiogenesis and glucose metabolism 43, 44, 45. Mechanistic studies revealed that Akt‐mediated cardiac protection is mainly attributed to the prevention of apoptosis and oxidative damage 24, 46, 47, 48. Matsui's group reported that up‐regulation of Akt activity significantly protected cardiomyocytes from apoptosis in response to hypoxia *in vitro* 49. Moreover, improvement of Akt activity significantly limited infarct size after ischemia/reperfusion injury and ameliorated doxorubicin‐induced cardiac dysfunction as a result of the inhibition of apoptosis 50, 51. Also, garlic is reported to lower cardiac oxidative stress *via* activation of the PI3K/AKT/Nrf2 pathway in diabetic rats 24. Additionally, hemin decreases cardiac oxidative stress in a rat model of systemic hypertension *via* PI3K/Akt signalling 52. We noted that activation of the PI3K/Akt/GSK3β signalling pathway induced by LDR in the diabetic hearts occurred in a dose‐dependent manner, so whether activation of Akt signalling itself was a component of LDR‐induced cardiac protection against diabetic cardiomyopathy was investigated *in vitro*. Primary cardiomyocytes were treated with HG/Pal to mimic type 1 diabetes *in vitro* and these cells were then treated with/without Akt siRNA followed by exposure to LDR. Both 25 and 50 mGy of LDR equally induced protection against diabetic cardiomyopathy. Thus, 25 mGy was chosen as a minimum effective, but more safer dose in the *in vitro* study. Data show that Akt knockdown strongly suppressed LDR‐induced prevention of cardiomyocyte hypertrophy and fibrosis associated with suppression of LDR‐induced anti‐apoptotic and anti‐oxidant effects in HG/Pal‐treated cardiomyocytes. Therefore, we demonstrated for the first time that the activation of Akt‐mediated anti‐apoptotic and anti‐oxidant functions contributes to LDR‐induced cardiac protection against diabetes.

How Akt mediates LDR's cardiac protection was not certain, so we approached the next studies with the understanding that activation of Akt/MDM2/P53 signalling always leads to anti‐apoptotic effects and that activation of Akt/nrf2 signalling always leads to anti‐oxidant effects 19, 53. Our *in vivo* study confirmed that LDR at medium or high dose significantly reduced activation of P53, an upstream inducer of apoptosis associated with increased expression of cardiac MDM2, a negative regulator of P53. Meanwhile enhanced Nrf2 nuclear translocation in LDR‐treated diabetic hearts was also observed associated with the increase in the expressions of multiple anti‐oxidants. Whether the above signallings mediate LDR‐induced cardiac protection against diabetes was also investigated in the current study by knockdown of MDM2 and Nrf2 respectively. Data show that the knockdown of MDM2 significantly enhanced P53 activity and subsequent apoptosis which impaired LDR‐induced prevention of cell hypertrophy and fibrosis in HG/Pal‐treated cardiomyocytes. Similarly, knockdown of Nrf2 also suppressed LDR‐induced anti‐oxidant effects and cardioprotection. Therefore, we concluded that LDR prevented diabetic cardiomyopathy likely because of the inhibition of apoptosis *via* the activation of the Akt/MDM2/P53 and inhibition of oxidative stress *via* Akt/nrf2 signalling pathways. Additionally, we also confirmed that LDR at the dose of 25 and 50 mGy remarkably lowered the blood glucose levels of diabetic mice. Whether the hypoglycaemic effect of LDR indirectly contributed to LDR‐induced prevention on diabetic cardiomyopathy still needs further investigation.

Ionic radiation at high dose is considered harmful, leading to DNA damage, cytotoxicity and tumorigenesis 11, 54, 55, 56. Although evidence suggests that exposure to LDR induced multiple beneficial effects, especially in diabetes 10, 12, 13, 14, whether there is a potential risk of fatal malignancy related to LDR has been frequently discussed but no conclusion is available at this time. Epidemiological surveys indicate that individuals exposed to less than 100 mGy had no increase or reduced risk of solid‐cancer incidence; no increase in leukaemia; no increase in cardiovascular diseases and perhaps had increased longevity. Also, no medications used in clinical practice are absolutely nontoxic. In the present study, we investigated a series of classic cancer markers levels in the plasma of mice among groups, the results showed that the plasmatic levels of all these markers of LDR‐treated mice were comparable to either healthy or diabetic mice, indicating the dose of LDR selected in this study were safe. Therefore, there is a need to evaluate the application of LDR to be realistic about its use and to understand whether it has a critical role in the prevention of diabetic complications.

In summary, diabetic cardiomyopathy is often an eventually fatal complication for diabetic patients who have cardiac fibrosis, hypertrophy and cardiac dysfunction followed by severe heart failure. Diabetes‐induced apoptosis, and oxidative stress are thought to be mediators of this pathology so a strategy that simultaneously suppresses these events may be ideal for prevention of diabetic cardiomyopathy. Our previous work indicates that LDR prevented cardiac damage at early stages of diabetes and this was attributed to inhibition of inflammation. Additionally, in this study, we confirmed that LDR prevented diabetic cardiomyopathy at the late‐stage of diabetes and this was because of suppression of diabetes‐induced apoptosis and oxidative stress *via* the Akt‐mediated MDM2/P53 pathways and the Nrf2/keap1 pathway respectively.

## Conflicts of interest

The authors confirm that there are no conflicts of interest.

## Supporting information


**Figure S1** Effect of LDR on hyperglycaemia in diabetic mice.
**Figure S2** LDR prevented diabetes‐induced pathological changes and fibrosis in the heart tissue.
**Figure S3** Effect of LDR on Nrf2‐mediated anti‐oxidant protein expression.
**Figure S4** LDR prevented diabetes‐induced inactivation of the PI3K/Akt/GSK3β signalling pathway.
**Figure S5** Effects of LDR on Nrf2 nuclear translocation, P53 activity and activation of the PI3K/Akt/GSK3β signalling pathway.
**Data S1** Supplementary materials and methods.
