# Supplementary material for: Low‐dose radiation prevents type 1 diabetes‐induced cardiomyopathy via activation of AKT mediated anti‐apoptotic and anti‐oxidant effects
Source: J Cell Mol Med. 2016 Mar 15;20(7):1352–66. doi: 10.1111/jcmm.12823 (PMC4929303; doi:10.1111/jcmm.12823)
Supplement: Supplementary file 1 — Figure S1 Effect of LDR on hyperglycaemia in diabetic mice. Figure S2 LDR prevented diabetes‐induced pathological changes and fibrosis in the heart tissue. Figure S3 Effect of LDR on Nrf2‐mediated anti‐oxidant protein expression. Figure S4 LDR prevented diabetes‐induced inactivation of the PI3K/Akt/GSK3β signalling pathway. Figure S5 Effects of LDR on Nrf2 nuclear translocation, P53 activity and activation of the PI3K/Akt/GSK3β signalling pathway. Data S1 Supplementary materials and methods. [file JCMM-20-1352-s001.docx]

**Supplementary Materials and Methods**

***Establishment of type 1 diabetic mouse model***

Male C57BL/6J mice eight weeks-of-age were purchased from the Experimental Animal Center of Beijing University (Beijing, China). Animals were allowed to acclimate for 2 weeks at 22 °C with a 12:12-h light-dark cycle and free access to rodent chow and tap water. We induced type 1 diabetes by fasting animals overnight and then administering STZ (50 mg/kg/day for 5 consecutive days). Control mice received an equivalent volume of citrate buffer. Five days after the last STZ injection, blood glucose level was measured with a FreeStyle complete blood glucose monitor (Abbott Diabetes Care, Alameda, CA). Mice were considered diabetic when blood glucose levels > 12 mmol/L.

***Cardiomyocyte isolation, culture and treatments***

Cardiomyocytes were isolated as described [[1-3](#_ENREF_1)]. Mice (2 months-of-age) were anesthetized with ketamine (43.5 mg/kg, im), acepromazine (1.5 mg/kg), and xylazine (1.7 mg/kg), and given heparin (100 U/ml). After median sternotomy,  the heart was quickly excised and arrested in ice-cold Ca^2+^-Tyrode solution. Hearts were perfused in the Langendorff mode on a gravity flow system at 37°C. Firstly hearts were perfused with a Ca^2+^-Tyrode solution aerated with 100% O_2_ (in mM: 137 NaCl, 5.4 KCl, 1.8 CaCl_2_, 0.5 MgCl_2_, 10 HEPES, 10 glucose, pH 7.4) to free the residual blood. After 5 min hearts were perfused with Ca^2+^-free Tyrode solution (in mM: 135 NaCl, 4 KCl, 1 MgCl_2_, 10 HEPES, 0.33 NaH_2_PO_4_, 10 BDM, 10 glucose, pH 7.2) for 3 min. Subsequently, hearts were digested by collagenase II (0.6 mg/ml) and pancreatin (0.08 mg/ml) dissolved in Ca^2+^-free Tyrode until a sudden increase in flow rate occurred, indicating effective tissue digestion. Then the heart tissue was removed, shredded, and filtered through a 140-µm nylon mesh. The supernatant was transferred to another tube filled with Krebs–Henseleit buffer (in mM: 0.5 EDTA, 5.1 KCl, 0.6 MgSO4, 118 NaCl, 1.2 KH2PO4, 10 glucose, 1 NaHCO3, 10 HEPES, 2 mg/ml BSA Fraction V, Sigma).and CaCl_2_ was added incrementally at 4-min intervals (five total steps) to increase Ca^2+^ concentration to 500 µmol/l. The suspension was then plated on laminin-coated culture (Dulbecco’s modified Eagles medium with glucose at the concentration of 5.5 mmol/l, 10% FBS, 1% penicillin and 1% kanamycin)for 5 h at 37 °C in a 5% CO_2_ incubator. Media was replaced and cultured for another 5 h before experimentation to wash away unattached cells and ensure that only rod-shaped myocytes were used for subsequent studies (totally 10 h before formal experiment). The cardiomyocytes were transfected with either negative control sense siRNA or target siRNA using Lipofectamine TM 2,000 (Invitrogen, Carlsbad, CA) transfection reagent for 48 h as described by the manufacturer. Thenthe cardiomyocytes were exposed to D-glucose (27.5 mM was added to reach the ﬁnal concentration 33 mM; high glucose, HG) for 24 h and palmitate (Pal, 62.5 μmol/L) was added during the last 15 h (totally 24 h for HG/ Pal treatment). Since as a saturated fatty acid, palmitate can not be directly absorbed by cells. Bovine serum albumin (BSA) acts as a carrier which can conjugated with palmitate and carries it into the cell for metabolism. Therefore, cell culture media with 2% bovine serum albumin (BSA, Sigma-Aldrich) was used during this period.

***Whole-body LDR in both mice and cardiomyocytes***

Diabetic and control mice were randomly divided into 2 groups respectively. One group was exposed to LDR (control/LDR; Con/LDR or diabetes/LDR; DM/LDR), and another group was given sham exposure (control or DM). Each group contains 8 mice. A 180-kVp X-ray generator (Model XSZ-Z20/20, China) was applied to deliver radiation at 12.5 mGy/min (120 kv, 13 mA). LDR was applied to the whole body of the animal every other day at 12.5, 25, or 50 mGy for 12 weeks. HG-treated cardiomyocytes received LDR at 25 mGy (12.5 mGy/min) every 6 h. The first radiation exposure occurred immediately after normal culture medium was replaced by HG medium.

***Echocardiography***

Transthoracic echocardiography (Echo) using a high-resolution imaging system for small animals (Vevo 770, VisualSonics, Canada), equipped with a high-frequency ultrasound probe (RMV-707B, VisualSonics) was performed for all anesthetized animals. All hair was removed from the animal chest and an aquasonic clear ultrasound gel (Parker Laboratories, Fairfield, NJ) without bubbles was applied to the surface of the thorax to optimize cardiac chamber visibility. Parasternal long-axis and short-axis views were acquired. Ejection fraction percent (EF%), fractional shortening percent (FS%), LV end-diastolic diameter (LVID;d), LV end-systolic diameter (LVID;s), left ventricular posterior wall thickness in end-diastole (LVPW;d), left ventricular posterior wall thickness in end-systole (LVPW;s), interventricular septal thickness in end-diastole (IVS;d), interventricular septal thickness in end-systole (IVS;s), LV mass and corrected LV mass were calculated using Vevo 770 software. Data are average values of 10 cardiac cycles [[4](#_ENREF_4),[5](#_ENREF_5)].

***Morphological examination of cardiac myocardium***

Mice were sacrificed, and hearts and tibias were removed and washed in cold saline. Heart weight and tibial lengths were noted. Additionally, myocardial tissue was isolated and fixed with 10% formalin for two days at room temperature. After dehydration in ethanol, tissue blocks were embedded in paraffin, cut 3 μm thick sections and stained with hematoxylin and eosin (H&E) for general morphological examination [[6](#_ENREF_6)]. Cardiac ﬁbrosis was quantified with 0.1% Sirius-red F3BA and 0.25% Fast green FCF for collagen accumulation, as described [[7](#_ENREF_7)]. Collagen was measured by quantitative analysis of the Sirius-red positive area using Image Pro software (Media Cybernetics, Silver Spring, MD).

***Terminal deoxynucleotidyl transferase-mediated dUTP nick end labeling (TUNEL) staining***

For TUNEL staining, slides were stained with the ApopTag peroxidase *in situ.* An apoptosis kit (Chemicon, Temecula, CA) was used to measure cell death [[8](#_ENREF_8)]. After deparaffinizing and rehydrating, each slide was treated with proteinase K (20 mg/L) for 20 min. Endogenous peroxidase was inhibited with 3% hydrogen peroxide for 5 min, and then incubated for 1 h with the TUNEL reaction mixture containing terminal deoxynucleotidyl transferase (TdT) and digoxigenin-11-dUTP. The TdT reaction was carried out in a humidified chamber at 37 °C, and 2XSSC was applied, and the mixture was incubated in the dark for 15 min. Counterstaining was performed with 4',6-diamidino-2-phenylindole (DAPI). For negative controls, TdT was omitted from the reaction mixture. Apoptosis was measured by counting TUNEL-positive cells selected randomly from 10 fields at ×40. Results are TUNEL-positive cells per 10^3^ cells.

***Caspase-3 activity***

Caspase-3 activation was measured as described (14). Briefly, fresh heart tissues were homogenized with Telfon homogenizer in an extract buffer, and homogenate was centrifuged at 20,000 × g for 30 min. Supernatant was diluted with an assay buffer and incubated at 37 °C with 200 μmol/l caspase-3 substrate I (Ac-DEVD-pNA [N-acetyl-asp-glu-val-asp-pNA], CalBiochem, La Jolla, CA). Substrate cleavage was monitored at 405 nm with a microplate reader.

***Lipid oxidation***

A thiobarbituric acid (TBA) assay was used to measure relative malondialdehyde (MDA) production as an index of lipid peroxidation [[9](#_ENREF_9)]. Briefly, tissue proteins were collected by centrifugation at 12,000 × *g* at 4 °C for 15 min, and protein was measured with Bradford assay. Then, 50 μl of sample was mixed with 20 μl of 8.1% SDS, 150 μl of 20% acetic acid, and 210 μl of 0.0571% TBA, and incubated at 90 °C for 70 min. Samples were centrifuged at 4,000 rpm for 15 min at 4 °C, harvested, transferred to 96-well plates, and optical density was read at 540 nm. Data are expressed as nmol/mg protein.

***ROS generation***

ROS in cardiomyocytes were measured using a kit from Cell Biolabs (San Diego, CA). Brieﬂy, cells were incubated with the peroxide-speciﬁc probe 2’,7’-dichloroﬂuorescein diacetate for 1 h followed by LPS stimulation for 1 h. Fluorescent signals were measured with a ﬂuorescent reader.

***Nuclei isolation***

Nuclei of the cardiomyocytes from both *in vivo* and *in vitro* studies were isolated using nuclei isolation kit (NUC- 201, Sigma, MO, USA) as previously [[10](#_ENREF_10)]. Briefly, 50 mg caridac tissues or 1 x 10^7^ cardiomyocytes were homogenized for 50 sec. within 300 ml cold lysis buffer containing 1 ml dithiothreitol (DTT) and 0.1% Triton X-100. After that, 600 ml cold 1.8 mol/L Cushion Solution (Sucrose Cushion Solution: Sucrose Cushion Buffer: DDT = 900: 100: 1) was add to the lysis solution. The mixture was transferred to a new tube pre-loaded with 300 ml 1.8 mol/L Sucrose Cushion Solution followed by a centrifugation at 30,000 x g for 45 min. The supernatant containing cytoplasmic component was saved for later analysis. Nuclei were visible as thin pellet at the bottom of tube.

***siRNA transfection***

Primary cardiomyocytes were transfected with either negative control sense siRNA ( Invitrogen, Carlsbad, CA) or mouse Akt antisense siRNA (sense: 5’-CCGGUGCUACUUCCUCAAGAACGCU-3’; anti-sense: 5’-AGUUGCAAUAGCUACUUCCUUUCCA-3’), mouse MDM2 antisense siRNA (sense: 5’-GCAACUCUAACUCUCCCAGUGAGUA-3’; anti-sense: 5’- UUCGCACGGGGUCAGUAAGGUUCCC-3’) and mouse Nrf2 antisese siRNA (sence: 5’- ACGCAUUAGACGCAACUUUAAAUUC-3’; anti-sense: 5’-AACCUUACUCUUCGCUCGCCUGCGG-3’) using Lipofectamine TM 2,000 (Invitrogen, Carlsbad, CA) transfection reagent for 48 h as described by the manufacturer. Transfection was followed by treatment with HG/Pal with/without exposure to LDR at 25 mGy as indicated.

***Western blotting assay***

Cardiac tissues were homogenized in lysis buffer (Santa Cruz Biotechnology, Santa Cruz, CA) and the supernatants were collected by centrifugation at 12,000 × g and 4 °C. Equal amounts of protein from each sample were separated on 10% SDS-PAGE and transferred to nitrocellulose membranes. After blocking with non-fat milk for 1 h at room temperature, membranes were incubated overnight at 4 °C with the following primary antibodies: atrial natriuretic peptide (ANP, 1:1,000), brain natriuretic peptide (BNP, 1:1,000), β-myosin heavy chain (β-MHC, 1:1,000), connective tissue growth factor (CTGF, 1:2,000), transforming growth factor-β (TGF-β, 1:1,000), murine double minute 2 (MDM2, 1:1,000), B-cell lymphoma-2 (BCL-2, 1:1,000), Bcl-2 Associated X Protein (BAX, 1:1,000), Mouse double minute 2 homolog (MDM2, 1:1,000), 3-Nitrotyrosine (3-NT, 1:2,000), 4-Hydroxynonenal (4-HNE, 1:1,000), Nuclear factor (erythroid-derived 2)-like 2 (Nrf2, 1:1,000), heme oxygenase-1 (HO-1, 1:1,000), NADPH: quinone oxidoreductase 1 (NQO-1, 1:1,000), catalase (CAT, 1:1,000), superoxide dismutase-1 (SOD-1, 1:1,000) and β-actin (1:1,000), which were purchased from Abcam (Cambridge, MA). Phosphorylated-P53 (p-P53, 1:1,000), total-P53 (t-P53, 1:1,000), cleaved-caspase-3 (C-cas3, 1:500), phosphorylated-protein kinase B (p-AKT, 1:1,000), total-AKT (1:1,000), phosphorylated-glycogen synthase kinase-3β (GSK-3β, 1:1,000), total-GSK-3β (1:1,000), phosphorylated-Fyn (1:1,000) and total-Fyn (1:1,000) were purchased from Cell Signaling Technology (Danvers, MA). After three washes in Tris-buffered saline containing 0.05% Tween 20 (TBST), the membranes were incubated with horseradish peroxidase-conjugated secondary antibodies for 1 h at room temperature. Antigen-antibody complexes were then visualized using an enhanced chemiluminescence kit (Amersham, Piscataway, NJ), and the intensity of the protein bands was quantified using Quantity one software (Version 4.6.2, Bio-Rad).

***RNA isolation and real-time quantitative polymerase chain reaction (RT-PCR)***

Total RNA was extracted from heart tissues or cardiomyocytes using Trizol reagent (RNA STAT 60 Tel-Test, Ambion, Austin, TX). RNA concentration and purity were quantified using a Nanodrop ND-1,000 spectrophotometer (Thermo Scientific, Wilmington, DE). Two μg total RNA was reversely transcribed using an avian myeloblastosis virus reverse transcriptase kit (Promega, Madison, WI) following the manufacturer’s protocol. Primers (mouse *ho-1*: Mm00840165_g1; mouse *nqo-1*: Mm1253561_m1; mouse *cat*: Mm00437992_m1; mouse *sod-1*: Mm01344233_g1 and mouse β-Actin: Mm00607939_s1) for PCR were purchased from Applied Biosystems (Carlsbad, CA). TaqMan Universal PCR Master Mix (Applied Biosystems) was used to prepare the PCR mix. The amplification reactions were carried out in a 20 μl reaction system that included the following: TaqMan Universal PCR Master Mix 10 µl, primers 1 µl, cDNA 9 µl and performed in duplicate for each sample the ABI 7300 Real-Time PCR system (Life Technologies Corporation, Carlsbad, CA) with initial hold steps (50 °C for 2 min, followed by 95 °C for 10 min) and 50 cycles of a two-step PCR (92 °C for 15 sec and 60 °C for 1 min). The fluorescence intensity of each sample was measured at each temperature change to monitor amplification of the target gene. The comparative cycle time method was used to measure fold-differences between samples. The comparative CT method quantified the amount of target, normalized to an endogenous reference (β-actin) and relative to a calibrator (2^-∆∆Ct^).

**References**

1. **Luo J, Hill BG, Gu Y, et al.** Mechanisms of acrolein-induced myocardial dysfunction: implications for environmental and endogenous aldehyde exposure. *Am J Physiol Heart Circ Physiol*. 2007; 293: H3673-84.

2. **Sambrano GR, Fraser I, Han H, et al.** Navigating the signalling network in mouse cardiac myocytes. *Nature*. 2002; 420: 712-4.

3. **Pinz I, Zhu M, Mende U, et al.** An improved isolation procedure for adult mouse cardiomyocytes. *Cell Biochem Biophys*. 2011; 61: 93-101.

4. **Basu R, Oudit GY, Wang X, et al.** Type 1 diabetic cardiomyopathy in the Akita (Ins2WT/C96Y) mouse model is characterized by lipotoxicity and diastolic dysfunction with preserved systolic function. *Am J Physiol Heart Circ Physiol*. 2009; 297: H2096-108.

5. **Zhang C, Huang Z, Gu J, et al.** Fibroblast growth factor 21 protects the heart from apoptosis in a diabetic mouse model via extracellular signal-regulated kinase 1/2-dependent signalling pathway. *Diabetologia*. 2015; 58: 1937-48.

6. **Shao M, Lu X, Cong W, et al.** Multiple low-dose radiation prevents type 2 diabetes-induced renal damage through attenuation of dyslipidemia and insulin resistance and subsequent renal inflammation and oxidative stress. *PLoS One*. 2014; 9: e92574.

7. **Zhou G, Li X, Hein DW, et al.** Metallothionein suppresses angiotensin II-induced nicotinamide adenine dinucleotide phosphate oxidase activation, nitrosative stress, apoptosis, and pathological remodeling in the diabetic heart. *J Am Coll Cardiol*. 2008; 52: 655-66.

8. **Cai L, Wang Y, Zhou G, et al.** Attenuation by metallothionein of early cardiac cell death via suppression of mitochondrial oxidative stress results in a prevention of diabetic cardiomyopathy. *J Am Coll Cardiol*. 2006; 48: 1688-97.

9. **Cai L, Wang J, Li Y, et al.** Inhibition of superoxide generation and associated nitrosative damage is involved in metallothionein prevention of diabetic cardiomyopathy. *Diabetes*. 2005; 54: 1829-37.

10. **Zhang C, Lu X, Tan Y, et al.** Diabetes-induced hepatic pathogenic damage, inflammation, oxidative stress, and insulin resistance was exacerbated in zinc deficient mouse model. *PLoS One*. 2012; 7: e49257.

**Supplementary Figures**

**
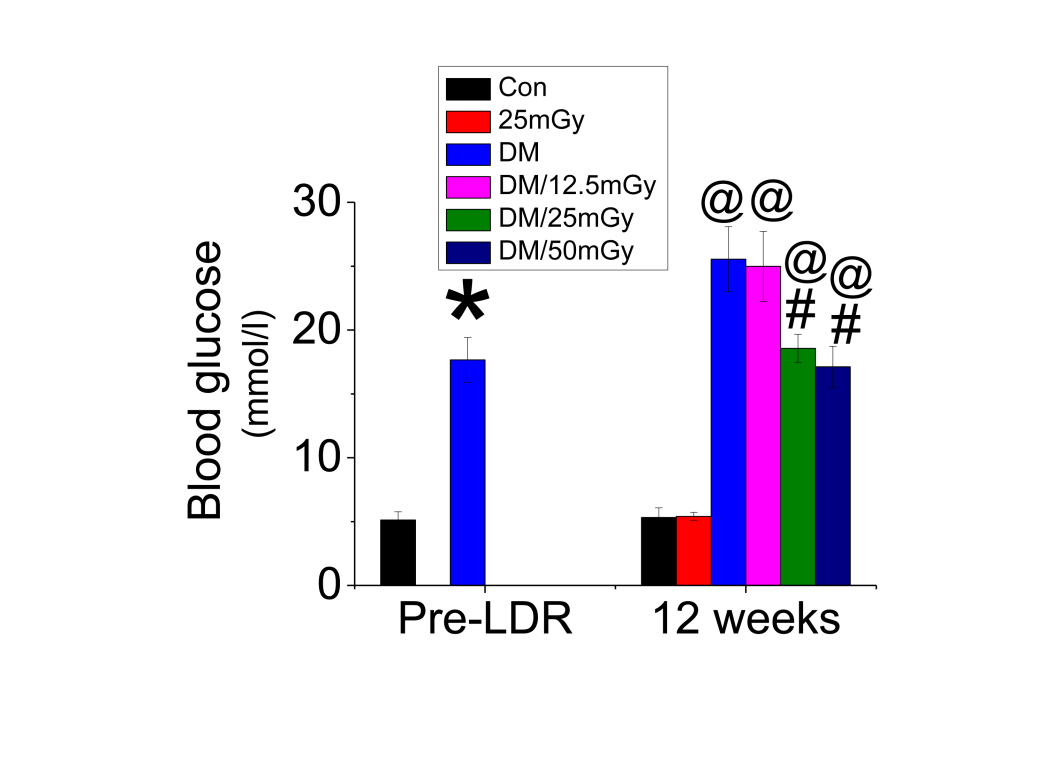
**

**Supplemental Figure S1. Effect of LDR on hyperglycemia in diabetic mice.** Mice were received multiple low-dose STZ injection (50 mg/kg for 5 days) . Five days after the last injection, the blood glucose levels were examined. Hyperglycemia was diagnosed once the blood glucose > 12 mmol/L. After that the mice in non-diabetic and diabetic group were received whole-body LDR treatment for 12 weeks. At the end-point the blood glucose levels were also examined in each group.


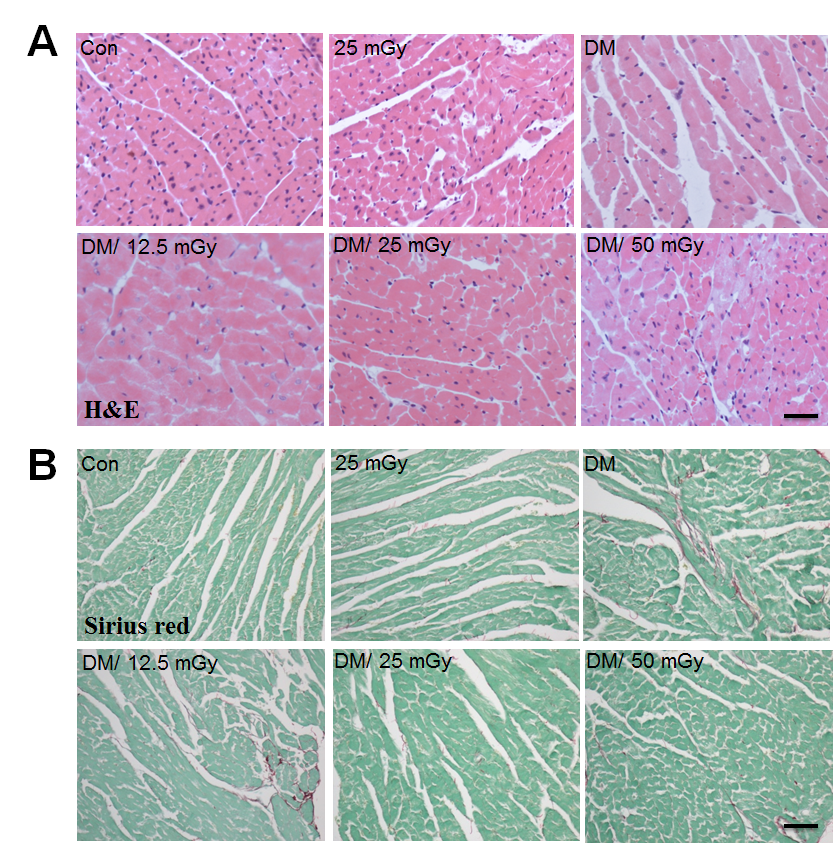


**Supplemental Figure 2. LDR prevented diabetes-induced pathological changes and fibrosis in the heart tissue.** Representative images of hematoxylin and eosin (H&E; A), and Sirius-red staining (B) for visualizing renal pathological changes, and collagen deposition, respectively. 40x magnification.


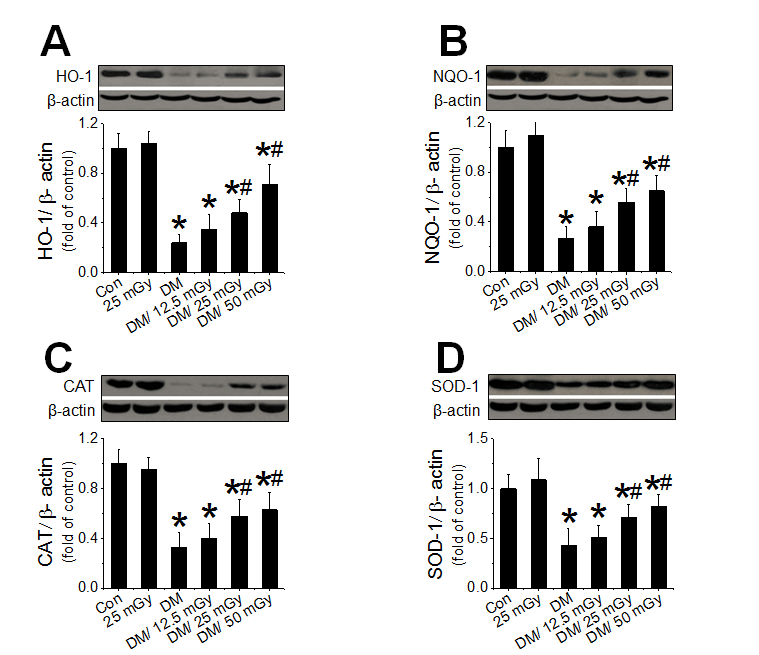


**Supplemental Figure S3. Effect of LDR on Nrf2-mediated anti-oxidant protein expression.** Expressions of Nrf2-mediated downstream target anti-oxidant protein including HO-1 (A), NQO-1 (B), CAT (C) and SOD-1 (D), in hearts of each group were measured with Western blot. Data are presented as means ± SD, n = 8/ group. **P* < 0.05 *vs*. the Con group; ^#^*P* < 0.05 *vs.* the DM group.


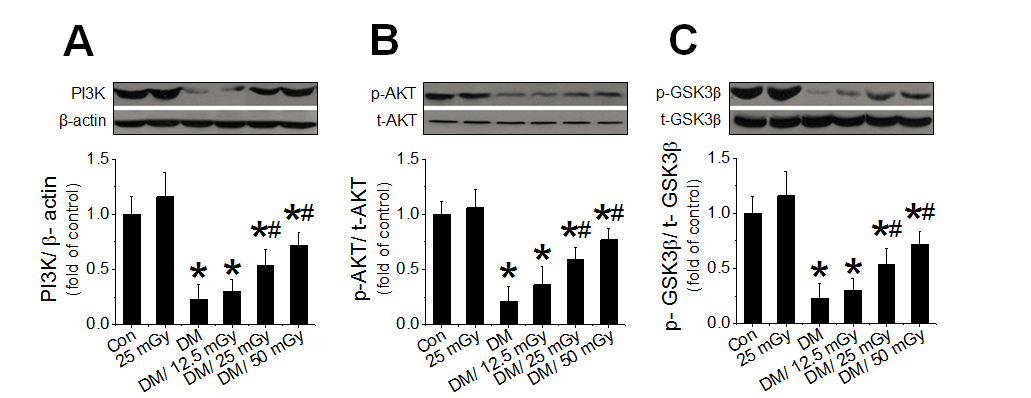


**Figure S4. LDR prevented diabetes-induced inactivation of the PI3K/Akt/GSK3β signaling pathway.** Western blot assay of cardiac tissue for PI3K (A) and phosphorylated and total AKT (B) and GSK-3β (C) were suppressed in diabetic hearts. Exposure to LDR at 25 or 50 mGy significantly reserved this signaling. Data are presented as means ± SD, n = 8/ group. **P* < 0.05 *vs*. the Con group; ^#^*P* < 0.05 *vs.* the DM group.


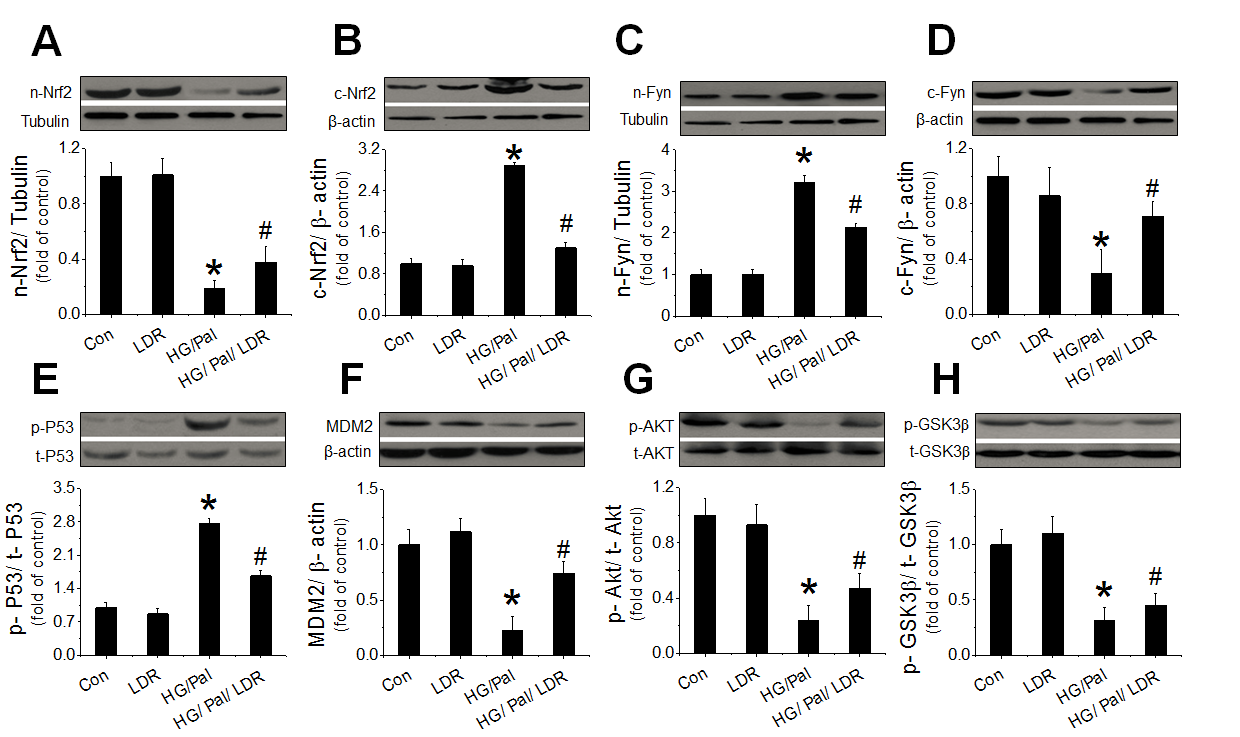


**Supplemental Figure S5. Effects of LDR on Nrf2 nuclear translocation, P53 activity and activation of the PI3K/Akt/GSK3β signaling pathway.** The translocation between the nuclei and the cytosol of Nrf2 (A&B) or Fyn (C&D) was evaluated by measuring protein of each of in the nucleus and cytosol, respectively. P53 phosphorylation (E), MDM2 expression (F), Akt, and GSK3β phosphorylation (G&H) were also quantified by Western blot. Data are presented as means ± SD, n = 8/ group. **P* < 0.05 *vs*. the Con group; ^#^*P* < 0.05 *vs.* the DM group.
